# Favourable outcome after 26 minutes of "Compression only" resuscitation: a case report

**DOI:** 10.1186/1757-7241-18-19

**Published:** 2010-04-16

**Authors:** Jon Erik Steen-Hansen

**Affiliations:** 1 Prehospital Clinic, Vestfold Hospital Trust and Telemark Hospital Trust, Box 2168, NO-3103 Tønsberg, Norway

## Abstract

**Case presentation:**

A 49 year old man had ventricular fibrillation in his home, at room temperature, due to an ST-elevation myocardial infarction. He received Cardiac compression only resuscitation (CC-only) for 26 minutes by his wife, followed by four minutes of standard CPR by other lay persons until EMS-arrival. Gasping and moaning were observed during most of the CC-only period. The ambulance arrived at 30 minutes. The first ECG showed a fine ventricular fibrillation. Restoration of spontaneous circulation (ROSC) was achieved at 49 minutes after a total of four defibrillatory shocks. The patient recovered without any cerebral damage, and was discharged to his home after eight days hospitalization.

**Conclusions:**

This case demonstrates that early and powerful cardiac compressions alone without rescue breaths may maintain sufficient circulation and gas exchange to prevent neurological damage for more than 25 minutes. This should be kept in mind for Emergency Medical Dispatch Centrals giving Pre-arrival instructions to bystanders.

## Background

Telephone CPR [1], Dispatch guided CPR or Pre-arrival instructions are terms of efforts by the dispatcher to motivate bystanders performing CPR until EMS arrival. There is a debate about the safety of giving CC-only for not CPR-trained lay people when the cause of arrest is cardiac. ERC Guidelines of 2005 [2], states that CC-only may be used if the rescuer is not able or is unwilling to give rescue breaths. For dispatcher instruction, a recommendation of four minutes CC-only followed by a compression-ventilation ratio of 100:2 was proposed in 2005 [3]. Some studies have shown better or equal effect of CC-only than traditional CPR on survival [4-6].

The Norwegian 2009 consensus for Dispatch guided CPR states that CC-only should be given for 10 minutes, before rescue breaths are given [7].

In December 2009, rescue breaths instructions for *cardiac caused arrests *were removed completely from the protocols at our Emergency Medical Communication Centre (EMCC) in Tønsberg, covering Vestfold and Telemark Counties. CC-only instructions should be given regardless of time axis.

There were several reasons for this decision. Median ambulance response times for the covered area (29 ambulances, 11 000 square kilometres and a population of 360 000), the possible confusion in and between EMCC and caller by switching of protocols during the pre-arrival instructions, and almost two decades of negative experience with existing rescue breaths instructions were decisive elements.

Within 14 days use of the new CC-only protocol, the following case presented.

## Case presentation

A 49 year old male suddenly lost consciousness, in front of his wife at 01:48 AM the first night in the New Year. This happened in door, at room temperature. Seconds earlier, he said he could not feel his heartbeat any more. The wife confirmed an immediate respiratory arrest and no other signs of life.

In addition, the patient's mother-in-law, were present in the house. The family lives in a remote mountain area. The nearest ambulance station is located 21 km away, the roads are narrow, winding and on this night also snowy and icy.

The wife phoned the medical emergency number 1-1-3 to the EMCC, explained the situation, exact localisation, handed the telephone over to her mother and started to give CC-only as instructed by the dispatcher.

According to EMCC and ambulance documentation, including sound and ECG files, the time line is presented in Table 1.

**Table 1:** Time line

Accumulated time h:mm:ss	Activity
0:00:00	Emergency call received

0:00:36	Exact localization documented

0:00:55	Unconsciousness confirmed

0:01:13	Instruction to open airway

0:01:20	Respiratory arrest confirmed

0:01:30	Instruction to give CC-only, without any rescue breaths

0:02:11	Certain respiratory efforts observed. Compressions paused shortly

0:03:40	Gasping and moaning between compressions could be heard in the phone

0:04:13	Dispatcher emphasizes that the compression rate should be powerful and at a frequency about 100/min

0:09:20	Efforts to find some close living persons to assist on the scene

0:25:30	Two adult persons arrived at the front door. Shortly afterwards they took over the CPR process with 30:2 compression: ventilation ratio

0:30:17	First ambulance arrived (outside the house)

0:31:50	A fine VF is documented, and the first defibrillator shock given

0:49:14	Sustained ROSC achieved after 4 defibrillatory shocks

1:20:31	12 channel ECG shows STEMI

1:21:20	Air ambulance arrived

2:51:20	Patient arrived at a regional centre for invasive cardiology

**Figure 1:**
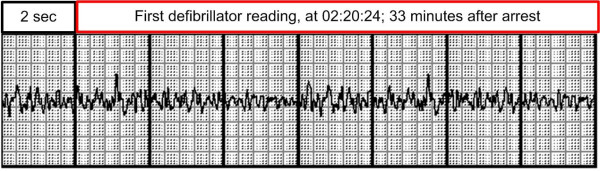
**First recorded ECG**.

During the first 26 minutes, CC-only, and no rescue breaths were given. This is well documented in the sound files, and confirmed by interview. The wife was encouraged by the fact that her husband gasped between compressions a great part of the time. The gasping ceased on two occasions during the 26 minutes. First, there was a short compression pause after the initial gasps, but then gasping ceased, and compressions were restarted. Secondly, some time later, the mother-in-law took over the compressions, to give the wife a little rest, but gasping ceased once more and the wife hurried to change back, with the result that gaspings again returned. The last four minutes before EMS arrival, standard CPR with chest compression and ventilation were performed by the patients son and a fourth person which had been called for. The first ambulance arrived with two paramedics and a general physician. A second ambulance arrived some minutes later. The ambulance teams had the impression that the first defibrillator reading was asystole, but the recordings shows a fine VF (Figure 1). They performed advanced CPR following protocols as best as they could. A semiautomatic biphasic defibrillator with a fixed 150 Joule setting was used and chest compressions for a mean of more than five minutes between each shock were performed. There were periods of organized rhythm after each shock. The airway was secured with a laryngeal tube. Intravenous adrenalin and a saline infusion were given. Ice packs were placed in the groin, armpit and neck region, to start therapeutic hypothermia.

**Figure 2:**
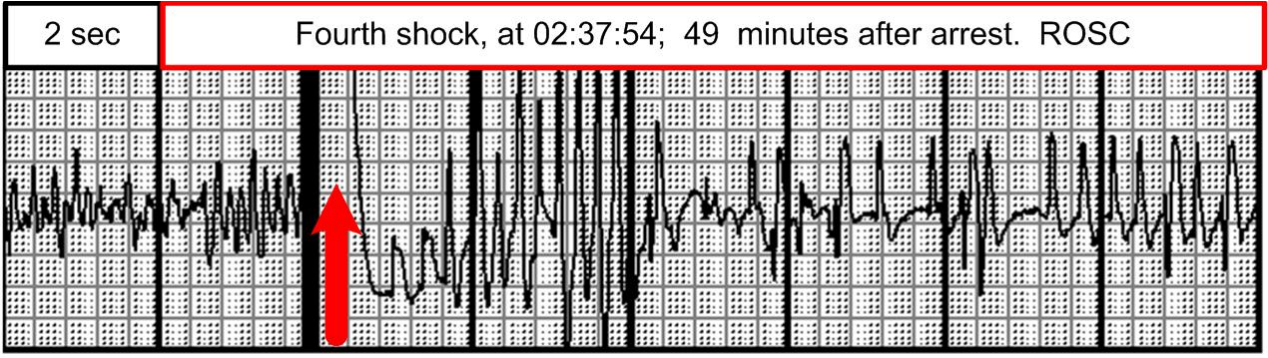
**The fourth defibrillatory shock and ROSC**.

**Figure 3:**
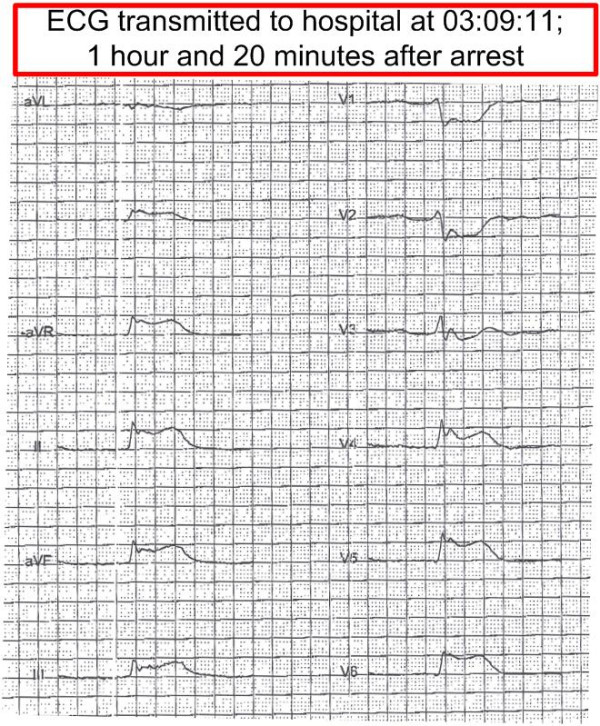
**12 channel ECG after ROSC**.

After ROSC (Figure 2) the respiratory movements increased in depth and frequency. The respiration was assisted for another five minutes. Then the patient vomited, causing laryngeal tube extubation, and he regained consciousness to a drowsy state. The patient had at day 14 some vague memories of these moments. A 12 channel ECG transmitted to hospital showed an anterior wall STEMI (Figure 3). It was then decided to transport the patient to an invasive cardiology centre with air ambulance.

During the flight, the helicopter physician administered tenecteplase as thrombolytic treatment because of long flight duration, and gave amiodarone because of an episode of VT.

**Figure 4:**
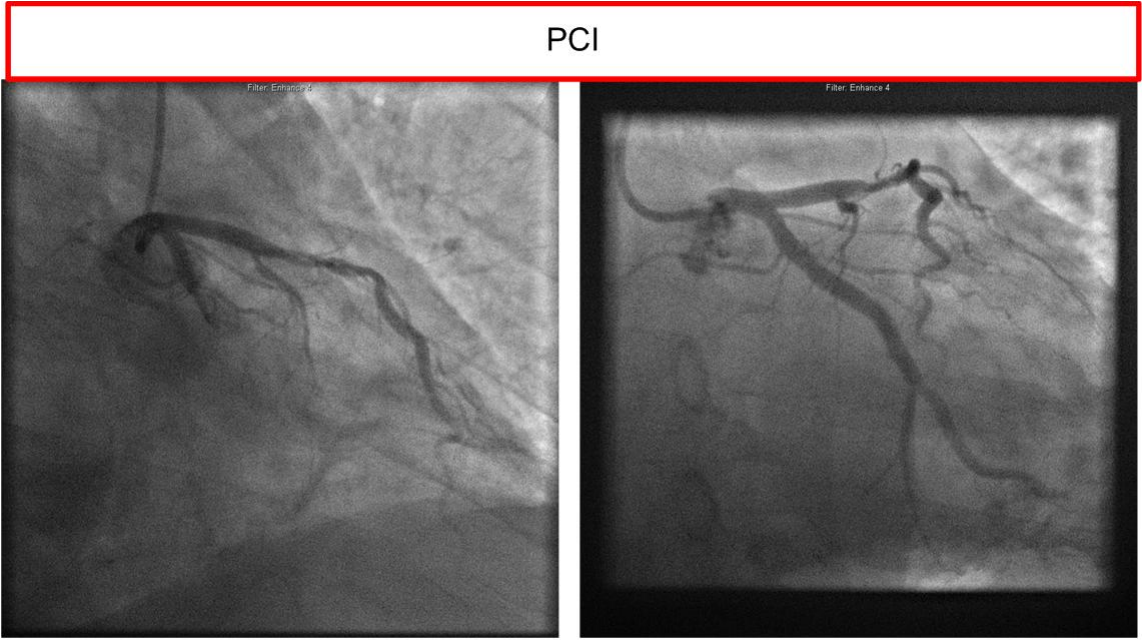
**PCI Images**.

At the hospital, the patient underwent a rescue percutaneous coronary intervention (PCI) with stent on the Circumflex coronary artery (Figure 4). He developed a moderate pneumonia, had one episode of bloody vomit, had multiple rib fractures, and some degree of flail chest, but ventilatory treatment was not necessary. Short time memory was initially reduced, but returned to normal from day five. He was discharged to his home on day eight. At 14 days he had a Modified Rankin score of zero, CPC of 1, (Examination by the author) and an OPC of 2. (Rib fractures and two remaining smaller coronary artery stenoses planned for treatment by a secondary PCI).

## Conclusions

This report demonstrates that if powerful cardiac compressions are started early, in this case less than two minutes after normothermic arrest, it is possible to maintain circulation and a sort of spontaneous respiratory movements resulting in gas exchange for more than 25 minutes. For this patient, this kind of respiration was sufficient for survival without neurological damage.

CC-only resuscitation without the time limits proposed until now may be kept in mind and taken in to consideration for Emergency Medical Dispatch Centres giving Pre-arrival instructions to bystanders.

## Abbreviations

CC-only: Cardiac compression-only resuscitation; CPC: Cerebral Performance Category; CPR: Cardio Pulmonary Resuscitation; EMCC: Emergency Medical Communication Centre; EMS: Emergency Medical System; ERC: European Resuscitation Council; OPC: Overall Performance Category; PCI: Percutaneous coronary intervention; ROSC: Restoration of spontaneous Circulation; STEMI: ST-elevation myocardial infarction; VF: Ventricular fibrillation; VT: Ventricular tachycardia.

## Competing interests

The authors declare that they have no competing interests.

## Consent

Written informed consent for publication as case report was obtained from the patient.
